# Enabling mental health task-sharing: a collective case study of undergraduate clinical associate training programmes in South Africa

**DOI:** 10.1186/s12909-022-03806-9

**Published:** 2022-10-28

**Authors:** Saiendhra Vasudevan Moodley, Jacqueline Wolvaardt, Christoffel Grobler

**Affiliations:** School of Health Systems and Public Health, University of Pretoria, Pretoria, South Africa

**Keywords:** Clinical associates, Task-sharing, Mental health, Psychiatry, Curriculum, Training, Assessment

## Abstract

**Background:**

There is a shortage of the human resources needed to deliver mental health services which is likely to be exacerbated by COVID-19. Due to mental health workforce shortages, task-shifting and task-sharing approaches have been implemented in a number of countries. Clinical associates, a mid-level cadre working under the supervision of medical practitioners, could play a role in delivering mental health services but it is not clear if they are adequately prepared. This study explored the mental health curriculum content of the undergraduate clinical associate training programmes in South Africa and the views of key informants of the adequacy of training in mental health.

**Methods:**

A qualitative collective case study approach was utilised for this multisite study at the three universities in South Africa offering clinical associate degrees. The study consisted of in-depth interviews utilising videoconferencing of individuals involved in each programme and a document review. Thematic analysis of the data was conducted.

**Results:**

Nineteen interviews were conducted. Mental health formed part of the curriculum in all three programmes with the bulk of the training taking place in the final year of the three-year degree. Facility-based training ranged from two weeks to four weeks with one university only using hospitals with mental health units while two universities used hospitals at which the students were based for the year regardless of potential mental health exposure they would receive. The list of curricula inclusions extended to seldom-seen conditions. The quality of training and supervision appeared site-dependant and only one university set minimum experiential targets.

**Conclusion:**

There is a basis on which to build the competencies and skills regarding mental health in this cadre. A training model that integrates mental health early in the undergraduate curriculum, focuses on common conditions and those with high disease burden, includes time in a mental health unit, provides facility-based trainers with detailed guidance to improve standardisation, and includes specific experiential targets that are monitored will enhance the potential utility of this cadre.

## Background

Mental and addictive disorders affect a substantial proportion of the world’s population and are major contributors to the global burden of disease [1]. It was estimated that a total of 162.5 million disability adjusted life years (DALYs) were due to mental and addictive disorders which is 6.8% of the total DALYs in 2016 [1]. In sub-Saharan Africa there was a 113.9% increase in total DALYs lost between 1990 and 2017 due to mental disorders [2]. Psychiatric epidemiological data for South Africa (SA) is limited, but the available data suggests that mental illness is a significant problem. The most recent nationally representative mental health data comes from the National Income Dynamics Study (NIDS) which only screens for depression [3]. In the fourth wave of the NIDS (2014–2015), 26% of adult participants were found to have significant depressive symptoms [3]. Suicide data provides an indication of mental illness burden [4, 5] and SA’s crude suicide mortality rate in 2016 was 11.6 per 100 000 population - higher than the global average of 10.6 per 100 000 population [6]. COVID-19 is likely to result in a rise in mental illness in SA as the pandemic and social distancing measures have resulted in stress, loneliness, and a reduction in social interactions which are known to increase the risk of mental illness [7].

There is a worldwide shortage of the human resources needed to deliver essential mental health interventions [8], and this shortage is a critical barrier preventing low- and middle- income countries from improving their mental health services [9, 10]. According to the World Health Organization [11], SA had 1.52 psychiatrists per 100 000 population in 2017. While this is above the global median, it falls short of the South African Society of Psychiatrists recommended target of 3.0 per 100 000 population [12]. It was reported that in 2019, 70% of psychiatrists in SA were servicing the small private sector [12]. As with the health workforce in general in SA, there is an urban-rural maldistribution of mental health human resources [13, 14].

Task shifting and task sharing approaches have been implemented in many countries in order to address mental health workforce shortages. More efficient use can be made of the health workforce by moving appropriate tasks to health workers who have fewer qualifications and have undergone shorter training [15]. The types of non-specialist health workers who deliver mental health services elsewhere include medical officers, nurses, and lay health workers [8, 16]. These workers have contributed to mental health services in various settings including clinics, community outreach services and halfway homes [8, 16]. The mental health tasks performed by these non-specialist health workers differ and depend on their level of training [8, 16]. These tasks include prevention, detection, and treatment of mental disorders [8, 16]. In a Cochrane systematic review assessing primary-level worker interventions in mental health, the authors found that there may be potential benefit to the use of primary-level workers in treatment of some adult mental disorders including depression and anxiety, depression related to pregnancy and childbirth, mental disorders in humanitarian settings, and severe mental disorders such as schizophrenia but further evidence is needed [17].

South Africa has extensive experience of using of task shifting and task sharing approaches in addressing its HIV epidemic including the use of nurses to initiate anti-retroviral therapy and lay health workers to deliver a number of health promotion interventions [18, 19]. Over the last decade, task sharing approaches have been increasingly explored as an option to deliver mental health services in South Africa [20]. Examples of these approaches include registered counsellors providing problem-solving therapy in the treatment of antenatal common mental disorders [21], a group-based Interpersonal Therapy intervention for depression delivered by lay HIV counsellors [22], and mental health counselling to patients with chronic disease(s) which was delivered by community health workers. [23] A study in five countries (including South Africa) found that the use of non-specialist health workers in mental health service delivery was perceived as acceptable and feasible by stakeholders if certain conditions are met including adequate training, the provision of ongoing supportive supervision, and adequate compensation.[24].

Clinical associates are a potential option to expand mental health services in South Africa. Clinical associates are mid-level health workers in the non-physician clinician category trained through a three-year Bachelor of Medicine in Clinical Practice (BMCP) degree at Walter Sisulu University and the Bachelor of Clinical Medical Practice (BCMP) degree at the University of Pretoria and University of Witwatersrand [25]. The first cohort of clinical associates entered the SA health system in 2011 [25]. The focus of clinical associate training in South Africa is the diagnosis and management of common medical conditions with the training preparing them to deliver services at district hospitals and at primary care level [26]. Their curricula use problem-based learning in the clinical context to apply their basic health sciences knowledge [26]. Similar cadres are found elsewhere in Africa (e.g. community health officers and community health extension workers in Nigeria, and clinical officers in Kenya and Uganda) though there is variation in the types of institutions doing the training and the quality of training [27]. South African clinical associates may have a greater depth of theoretical health sciences training as they complete a university degree. In general, this cadre of health worker in Africa provides diagnosis and treatment at primary healthcare facilities (clinics and health centres) and district hospital outpatient departments [27]. They are frontline health professionals who are the interface between patients and higher level care.

The available evidence suggests that SA has a high prevalence of mental disorders and these are a significant contributor to the disease burden and the COVID-19 pandemic is likely to exacerbate this. South Africa has a shortage of specialist mental health professionals with public-private sector and urban-rural maldistribution. Task sharing approaches are critical to ensure mental health service provision in underserved areas. The role of clinical associates in mental health service provision is currently ill defined and they are possibly an under-utilised resource in mental health task sharing approaches. While mental health does form part of their scope of practice [28], the extent or utility of the training is not clear. This study explored the mental health curriculum content of the undergraduate clinical associate training programmes in SA and the views of key informants of the adequacy of training in mental health.

## Methods

### Study design

A collective case study approach was utilised. According to Creswell and Poth[29], case study research is a qualitative approach that explores one or more contemporary, real-life bounded case or cases over time through in-depth data collection utilising multiple data sources. A collective case study is one in which multiple cases show different perspectives [29].

### Study setting

All the universities offering clinical associate degrees: Walter Sisulu University, the University of Pretoria and the University of Witwatersrand.

### Study population and sampling

Each of the clinical associate training programmes was considered a ‘case’. In-depth interviews were conducted with individuals involved in each programme. Purposive sampling was utilised. Participants were selected on the basis of being able to provide information on the mental health content of each programme. A BCMP academic co-ordinator from each university assisted in identifying the relevant individuals. For each programme, the following individuals were considered for inclusion:

- BCMP/BMCP academic co-ordinators.
- The individual(s) responsible for mental health teaching.
- The 2020 final year class representative.
- BCMP/BMCP site-based co-ordinators/supervisors at selected rotation sites.
- The clinicians providing mental health training at selected rotation sites.

### Measurement tools and data collection

Data were obtained from a combination of documents (such as study guides) and key informant in-depth interviews. BCMP/BMCP co-ordinators at the three universities were asked to provide the relevant documents. Interviews were conducted using videoconferencing. Participants were informed how data would be used/stored, and how privacy would be protected in line with Blackstone’s guidance [30]. The interview included questions on the aspects of mental health covered in the curriculum, the formal teaching in mental health, the practical training provided in mental health, and participant views on the adequacy of the mental health component of the curriculum and any gaps in the teaching. Interviews were audio recorded and hand-written notes were taken. The documents were reviewed to obtain data on the psychiatric disorders covered in the curriculum, training in the relevant procedures (skills) listed in the clinical associates scope of practice[28], training (if any) on mental health screening tools, and the types of assessments (oral/clinical/written) and their respective weightings for psychiatry component of the curriculum.

### Data management and analysis

The interviews were professionally transcribed and the documents and transcriptions were imported to Atlas.ti software. Codes were allocated to significant statements in the transcribed in-depth interviews and to significant information in the documents obtained [31] Data analysis used the approach outlined by Creswell and Poth [29] for case study research. The generated codes were then aggregated into categories and utilised to generate themes [29]. Direct interpretation (drawing meaning from a single instance) was employed [29]. Cross-case theme analysis was done to identify similarities and differences between the programmes and naturalistic generalisations related to what was learnt from the cases were developed[29].

### Processes to ensure quality of research

Frambach et al. [32] outlines various techniques that can be used in qualitative research to address the quality criteria of credibility, transferability, dependability and confirmability. Techniques used in this study were data triangulation (in-depth interviews of staff and students together with document review), iterative data analysis, peer debriefing, and maintaining an audit trail.

## Results

### Participants

A total of 19 interviews were conducted across the three universities offering the Bachelor of Clinical Medical Practice/Bachelor of Medicine in Clinical Practice(BCMP/BMCP) degree between 25 March 2021 and 29 July 2021. For each programme, a mixture of university-based and facility-based personnel were interviewed as well as the class representative of the previous (2020) graduating class. Eleven documents were reviewed. The number of interviews conducted and documents reviewed for each programme are shown in Table 1. In order to maintain confidentiality, the universities have not been named when presenting results but have been randomly designated as A, B or C.

**Table 1:** Interviews conducted and documents reviewed

	University A	University B	University C
**Interviews conducted**			
BCMP/BMCP Academic coordinators	4	1	2
Individuals responsible for mental health teaching at university	1		1
BCMP/BMCP training site-based co-ordinator/supervisor		4	3
Clinician providing mental health training at training sites	1		
2020 final year class representative	1	1	1
Total interviews	6*	6	7
**Documents reviewed**			
Study guides (or similar)	1	2	4
Rotation guide	1		
Preceptor manual	1		
Lecture slides	1		
Logbook		1	
Total documents	4	3	4

* One of the BCMP/BMCP academic co-ordinators was responsible for mental health teaching

### Timing of mental health in the curriculum

#### University A

In Year 1 some generic skills considered useful in mental health such as biopsychosocial assessment, communication and counselling are introduced. The mini-mental state exam is covered in their neurology block. In Year 2, they build on their counselling skills to include bereavement, grief and loss as well as substance use and have possible interaction with mental health patients during rotations e.g. emergency department and internal medicine. The formal mental health component of the curriculum takes place in Year 3 and includes both university-based teaching and facility-based practical training which has its drawbacks: *“I think that we integrate it a little bit too late, personally, because our students do start interacting with mental health patients already in the first year.”* (P10).

#### University B

At University B, there is no formal teaching related to mental health in Years 1 and 2. “*They will meet patients on the wards and in the casualty. But it is not one of the things that we teach them*” (P11). The view was that students would generally not be interested in mental health before the module which is in the third year:

“*You can teach someone, it doesn’t mean that people are going to learn that thing. So, they don’t learn to do it per se until they come to this module in which they have to do it, or they have to know, or they can’t pass the module…we don’t touch this topic until year three, they’re like, okay, I don’t need this now.”* (P15).

#### University C

University C introduces students to some aspects of mental health history taking, the mini-mental state examination, and the psychosocial (three-stage) assessment in Year 1. Communication skills and basic medical psychology are also included. Neurology is a theme (Year 2) where a few relevant aspects of psychiatry are touched on. Again, the bulk of mental health is included in Year 3. The lack of integration of into the earlier years comes at a cost:

*“…as a student you don’t do psych in first and second year. All of a sudden you’re doing psych in the final year, so you get surprised of all of the list of conditions that you have of psych, and you don’t know, you’ve never been exposed to any of them, you’ve never focused on any of them. So you don’t know which one is important and which one is of serious concern, which one is of emergency or what.”* (P19)

There was strong support that students should be formally introduced to some aspects of the mental health curriculum earlier in their programme. An earlier start would allow them to take advantage of the mental health learning opportunities in the wards and emergency departments and to integrate knowledge better. However, this would also need a change of mindset from students as noted by one interviewee: *“I do think sometimes students compartmentalise too much and they lose opportunities to learn, even though it’s not immediately relevant in terms of an upcoming exam”* (P17).

### Mental disorders included in the curriculum

The mental disorders that are included in the curriculum were extracted from the documents received (Table 2). The lists of disorders are fairly similar with all three lists including the common mental disorders viz. depressive disorders, anxiety disorders, and substance-related disorders. Schizophrenia is also included in all three curricula. An interviewee from University C raised a concern regarding the scope: “…*it’s basically the whole mental health book in there, all the topics, all the diseases, all the conditions are there, it’s just like how do you squeeze them in into short time?*” (P18).

Based on the interviews it is apparent that practical exposure to patients with these conditions varies significantly by site. It ranges from district hospitals without a psychiatric unit and exposure to only a limited number of these conditions/presentations (e.g. acute psychosis, suicidal behaviour) to those at a tertiary hospital with a broad range of the disorders.

**Table 2:** Conditions that form part of the curriculum as indicated in course, module and/or rotation guides

University A	University B	University C
Alcoholism	Acute substance intoxication	Attention-deficit hyperactivity disorder
Anorexia nervosa	Acute substance withdrawal	Alcoholism
Attention-Deficit/Hyperactivity Disorder	Alcoholism	Alzheimer’s
Autistic Disorder	Anxiety	Bipolar mood disorder (Type I and II)
Bereavement	Bipolar disorder	Conduct disorder
Bipolar disorder	Children’s behavioral disorder	Delirium
Bulimia nervosa	Delirium	Dementia
Conduct disorders	Dementia	Depression
Delusional disorder	Depression, acute	Family violence
Dependency, alcohol	Depression, chronic	Gender dysphoria
Dependency, drug	Drug addictions	Gender identity disorder
Domestic violence	Family violence	Generalized anxiety disorder
Dysthymic disorder	Mental health disorders related to female gender	Major depression disorder, chronic
Generalized anxiety disorder	Mental retardation	Mental retardation
Major depressive disorder	Psychiatric disorders in HIV	Obsessive compulsive disorder
Malingering	Psychosis, acute	Organic brain damage
Mental retardation	Psychosis, toxic	Personality disorders
Panic attack disorder	Schizophrenia and other chronic psychotic disorders	Phobias
Personality disorder	Substance abuse	Post-traumatic stress disorder
Phobias	Suicidal behavior	Psychosis, acute
Post-traumatic stress disorder		Psychosis, toxic
Psychoses, drug induced		Schizophrenia spectrum disorders, chronic
Schizophrenia		Substance abuse
Suicidal thoughts		Suicidal behaviour
Suicide attempt		
Withdrawal, alcohol		
Withdrawal, drug		

### Competencies and skills

The mental health (and related) competencies and skills planned for by each university were reviewed using both the documents and the interview data (Table 3). Based on this examination, all three universities include learning opportunities for all the elements of the mental health assessment. Universities A and C use electronic logbooks where students need to record patients seen and procedures done but do not specify minimum numbers. University B uses a manual logbook with minimum requirements e.g. “sedate five aggressive patients”.

**Table 3:** Competencies and skills covered as per documents reviewed and interviews conducted

	University A		University B		University C	
	**Documents**	**Interviews**	**Documents**	**Interviews**	**Documents**	**Interviews**
**Competencies**						
Mental health history	✔	✔	✔	✔	✔	✔
Mental status examination	✔	✔	✔	✔	✔	✔
Mini-mental state examination	✔	✔	✔	✔	✔	✔
Physical examination	✔	✔	✔	✔	✔	✔
Relevant bedside and laboratory investigations	✔	✔	✔	✔	✔	✔
Psychosocial or three-stage assessment	✔	✔	✔	✔	✔	✔
Diagnosis	✔	✔	✔	✔	✔	✔
Pharmacological management	✔	✔	✔	✔	✔	✔
Counselling	✔	✔	✔	✔	✔	✔
Suicide risk assessment*	**X**	**X**	**X**	**X**	**X**	**X**
Mental Health Care Act	✔	✔	✔	✔	✔	✔
72-hour observation	**X**	✔	**X**	✔	**X**	✔
Referral	✔	✔	✔	✔	✔	✔
Restraint	✔	✔	**X**	✔	**X**	**X**
Sedation	✔	✔	✔	✔	✔	**X**
Mental health promotion	✔	✔	✔	**X**	✔	**X**
**Skills**						
Communication	✔	✔	**X**	**X**	✔	✔
Information gathering (research)	**X**	**X**	✔	✔	✔	**X**
Patient notes and letters	✔	**X**	**X**	**X**	✔	✔
Teamwork	✔	✔	**X**	**X**	✔	**X**

* suicide risk assessment is included here as it was mentioned by a couple of participants as being important but there was no evidence that it was being covered

### University-based teaching

#### University A

There is theoretical block at the start of Year 3 on campus that includes one week of mental health which consists of formal lectures, videos and case-based learning. The teaching is currently done by one of the BCMP/BMCP co-ordinators who is a clinical associate. Psychiatrists from the local academic hospital only give input on the teaching material. Lectures given include mental health ethics and pharmacology of psychiatric drugs. A case-based approach is used to teach mental disorders with the theory being discussed before a case discussion. Extensive use is made of videos including a psychiatric interview:

“…. *so pre-Covid, in a classroom setting, we would watch the video together, I’d stop the video and then we would go through the mental status exam together, so that students could fully consult with that patient in the video as though they were doing it in real life. And then we’d diagnose the patient together, assess then according to DSM criteria, and then discuss the management around that patient …and that condition as a whole.*” (P03)

Due to the COVID-19 pandemic, contact teaching was limited. Students were given the videos to view on their own. Synchronous online sessions were held to discuss each of the conditions. On campus skills teaching was permitted and students attended a simulated ward session which included a simulated mental health patient that they had to assess and manage.

#### University B

There is a didactic period at the start of third year on campus in which seven themes are covered including mental health. Due to COVID-19 and political instability on campus, all mental health teaching shifted to the health facilities where students were based. Prior to that, it was unclear how much mental health content was taught during the didactic period, but one interviewee estimated 20 hours (done by the family physicians, medical officers, and clinical associates).

#### University C

A foundation week covering multiple clinical discipline in Year 3 includes a two-hour introductory lecture on mental health. They also receive a lecture in their third year on substance use and harm reduction. The mental health lecture is given by a psychiatrist based at the local academic hospital and was presented in a synchronous online format during the pandemic. The lecture includes an approach to mental health assessment, classification system, some interviewing skills and an overview of some common mental disorders. There was some concern expressed that this was not adequate: “*I’m slightly biased here because I think that it’s a very superficial overview that we give. And given the scope of mental health problems, you know, it’s probably not enough*.” (P06).

### Facility-based teaching and practical training

#### University A

There is a two-week mental health rotation in Year 3 to a psychiatric unit. Two of these units are in district hospitals, one is a regional hospital and the fourth is an academic hospital. The model used by University A is different from Universities B and C where the students have their mental health module at whichever hospital they are based for the year. One of the interviewees outline the rationale for University A’s different approach: “*…we’re looking to place our students in a location where there is a department, there is a consultant, where there is opportunity for students to really be engaged and learn.”* (P13).

The two-week mental health rotation at University A is shorter than the other specialties (generally four weeks), which was considered sub-optimal:

“*Never enough. Never, ever enough. I know every time I tell the preceptors it’s two weeks, they’re like that’s not nearly enough to do a psych rotation … But two weeks to grasp psychiatry and to grasp mental health, is definitely not enough time.”* (P10)

Each facility has a university-employed site facilitator who co-ordinates rotations but hospital-employed preceptors supervise and train the students. The preceptors are either psychiatric consultants or medical officers working in psychiatry unit. The students’ activities vary depending on the facility but generally consist of assessing mental health patients (in the wards, outpatient departments and/or emergency departments), attending patient rounds, and presenting patients to their preceptors. There is a preceptor manual provided by the university but it seems preceptors are left to their own devices to some extent as the training is:

“…*very site dependent, which becomes very difficult to get students to get to the same objectives because in the clinical setting there are some MOs* (medical officers) *who really love teaching and involve the clinical associate students in the ward round, in consultations, and students become quite comfortable with psych. Versus a hospital like* (the academic hospital), *they get a little bit lost and there’s medical students, there’s registrars, there’s interns, so the ward round doesn’t become as beneficial for them unless there’s something who’s a bit more inclusive in their teaching*.” (P03)

#### University B

Based on the documents, University B has a three-week facility-based mental health module in Year 3. However, interviewee responses varied as to whether the module at the facility was actually three or four weeks. This uncertainty was possibly due to time for the didactic campus-based component (which had not taken place in recent years) being added to time spent at the facility. There was a general concern whether this was sufficient:

“…*my main concern is that we spend only one month in these topics, only one time in three years, and one month. So it’s like, when the students go over these topics, or over this module, they don’t really go back again…it’s not like pneumonia they can keep seeing pneumonia every day, right? and studying through three years, and TB they study TB through the three years and several times.”* (P15)

The students do their mental health rotation at the hospital that they are based at. University B uses three district hospital and one regional hospital for training. Only the latter has a mental health unit. To maintain consistency, students are not placed in that mental health unit but follow a similar programme to students placed at facilities without a mental health unit. The rationale is that *“their scope of practice may not necessarily need them to be on the mental health unit…”* (P14). Despite attempts to standardise training at different sites, one interviewee did concede that “…*the amount of exposure they will have to patients who need mental health services varies by hospital site.*” (P11).

There are academic staff (“tutor-lecturers”) appointed by the university at each of the training facilities. These are either medical officers or clinical associates who are responsible for training across the specialties. Teaching consists of problem-based learning (PBL) and topic presentations. Students clerk patients with a mental health presentation, and present to the tutor-lecturer and class as part of PBL. The differential diagnoses, psychosocial assessment, and management plan is then discussed and learning needs identified. A standard list is used for the topic presentations. The student representative expressed some misgivings about the presentations:

*“…the whole thing of we present certain topics, this is also a bit of a problem sometimes, because we ourselves do the research, and it’s the first time we’re encountering this topic thoroughly, so you do your own research and everything…it doesn’t make it as effective as if maybe we had the lecture first.”* (P09)

The amount of practical mental health training appears to be limited. The students may or may not opportunistically encounter mental health patients in the emergency and outpatient departments and one participant acknowledged *“…it is possible for some students to go through the year, or to go through the course, without really handling mental health patients.*” (P11).

#### University C

Students at University C have a four-week mental health rotation in Year 3. The rotation is split into two weeks in the wards, one week in the emergency department and one week in the outpatients’ department at the hospital they are based. Fifteen (or in some years more) hospitals are utilised for training and range from district to tertiary levels and may or may not have a dedicated mental health unit. Some students spend a week in a community-based substance use programme instead of the outpatients’ department.

At each facility, there is an identified family medicine practitioner who is responsible for ensuring training but in practice students are often *“… siphoned off to somebody else who may or may be not be interested really in the training of clinical associates”* (P04). At some facilities, a psychiatrist or mental health nurse may be present to assist during the mental health rotation though it is generally medical officers. The students are expected to sit in on patient rounds and psychiatric interviews that are being done by the doctors as well as clerk and present mental health patients. The module guide indicates that students are required to have a “mental health longitudinal patient” with a chronic mental illness who they follow up for several months including doing a home visit. This aspect did not feature strongly in the interviews, so it is not clear how well this is implemented.

A key concern raised by interviewees was the variability in mental health training at the different sites and there was particular concern about students placed at rural facilities:

*“So, it varies a lot with respect to what they ultimately get exposed to during that four weeks…it really depends upon where they’ve been placed. And the majority of it, I think it varies, I mean, incredibly.”* (P04).

“*My own view, from the programme perspective, I think programme looks great, the way it’s structured. But in practice I don’t think they have enough support once they go off into the rural facilities*.” (P18).

The class representative stressed the importance of student agency in maximizing the benefit of the mental health and other rotations *“You have to, as an individual in (BCMP/BMCP), you have to be knowledge-driven, you need to, out of your own will look for patients that will give you the proper exposure you’re looking for”* (P05). She noted that doctors are then more likely to assist students with this attitude.

### Assessment

The assessments specifically for mental health at the three universities are shown in Table 4 along with other key features of the three programmes. At University A, mental health is also included in objective structured clinical examinations (OSCE). There are three OSCEs in the third-year and each usually have a mental health station e.g. a consultation with a simulated psychiatric patient. At the end of the third-year, the students at all three universities have a Clinical Associate National Examination (CANE) which includes mental health (blueprinted as 11% of the mark allocation of the written papers). The CANE consists of an Multiple Choice Question (MCQ) paper and a Modified Essay Question (MEQ) paper. All universities have independent (but similar) final OSCE exams. University A has a 12–14 station OSCE which usually has a mental health station. University B confirmed that a mental health station was included in their final OSCE the previous year. The final OSCE at University C usually includes at least one mental health station. There was some concern regarding assessments and examinations in general at University C as it was felt there was a lack of alignment between clinical practice and what they are assessed on. There was some acknowledgement of the missed opportunity of bedside assessment and the need for workplace-based assessments: “*I think we’ve been talking about workplace assessment, I think those are good…those are perhaps one of the ways forward.*” (P17).

.

#### University C

**Table 4:** Key features of mental health training in the three programmes

Programme	Formal lectures on campus (pre-COVID)	Facility-based teaching and training			Assessments
		**Duration**	**Site**	**Approach**	
University A	1 dedicated week	2 weeks	Hospital with a mental health unit	Practical	Patient report, ethics reflective journal (optional), preceptor observation of a clinical assessment, preceptor evaluation of overall performance, test consisting of multiple-choice questions (MCQ) and case-based questions
University B	20 h	3 weeks	Hospital where they are based for the year	Theoretical with limited (co-incidental) practical exposure	Patient-orientated medical record, topic presentations, problem-based learning discussions, test consisting of MCQ and MEQ
University C	3 h	4 weeks	Hospital where they are based for the year	Considerable variation between facilities	Patient report and presentation, assessments related to the longitudinal patient, a test, a skills assessment

### Training adequacy and competency to provide mental health services

#### University A

Besides issues linked to starting mental health training at a late stage of the programme and duration of the mental health rotation, other gaps noted by interviewees included inadequate theoretical component, a lack of exposure to child psychiatry and personality disorders, counselling at a “*very surface level*” (P10), and limited training on the substances that could be abused. There were differing views on whether the training as a whole was adequate and whether graduates are likely to be competent to deliver mental health services: *“So I don’t think it’s adequately covered. I think the clinical associates can add a lot of value to a mental health department and I think our curriculum doesn’t fully allow for them to see that, but it also doesn’t allow for us to fully go into the theory.”* (P03).

#### University B

The identified gaps in training included training related to childhood behavioural disorders, and dementia. There were differing views on whether mood disorders were a gap or not. One interviewee thought that students would struggle to manage mental health patients in the wards as they do not receive that exposure during their mental health training. There were differing views on training adequacy and competency to practice:

“*I believe it’s adequately covered, because they are prepared to do what is required in their scope of practice in terms of the 72-hour assessment, and then they refer the patients to the specialist. So we are actually covering well, that’s my view.*” (P14).

“*I will say that they can do emergency management, but even that one, at the time of graduation and going out there, they are not very comfortable with that yet. They’re not.*” (P11).

#### University C

The lack of emphasis on mental health teaching, minimal formal teaching and inadequate guidance as to the areas to focus on were identified as gaps at University C. One of the interviewees noted that “*… right now I think the message that we’re sending to our students based upon the way we teach the programme is that mental health is not that important, and it’s only important for four weeks out of your entire education.”* (P04) This sentiment was echoed by one of the site facilitators: *“So the exposure and opportunities are also playing the part there, but also the university itself doesn’t place enough emphasis or guide in terms of that.”* (P19).

Concerns were raised that students may not be exposed to mental health patients at some hospitals beyond the immediate management in emergency departments. It was noted that “…*a lot of their mental health knowledge, I think comes from them reading about it as opposed to actually experiencing those patients.”* (P04) A concern was raised about their diagnostic ability:

“*So, for instance, they may not necessarily be able to identify that somebody, say, has got a general anxiety disorder, or sometimes to some extent, even just depression. It’s very difficult for them to be able to pick up those nuances at the very beginning. And my view is that there ends up being a lot of misdiagnoses from them because they’re not trained adequately to do that.”* (P04)

There were varying responses on whether training adequately prepared clinical associates from this institution with respect to mental health with some reliance on reputation rather than evidence: *“I definitely think that if we passed the final OSCEs and the final exam papers, I feel like we all definitely have the necessary knowledge, because* (University C’s) *assignments were not easy. So if we got through I feel like we should trust the university and their level of assessment to tell us that we are competent.”* (P05).

While others reflected on the future utility of their graduates: “*If they are expected to manage patients on their own as, you know, quasi-medical practitioners, then I think a lot more work should go into training them to do it effectively.”* (P06).

## Discussion

The interviews and documents confirmed that mental health is a component of all three curricula. In all three programmes, virtually all mental health teaching and training occurs in the final year of the three-year degree. This design feature was considered a missed opportunity for earlier learning in other rotations. There was almost universal support for integrating mental health earlier in the curriculum. Given that individuals with chronic physical illness are at high risk for depression and anxiety and individuals with serious mental illness are at high risk for a number of chronic physical conditions [33], it would be ideal to integrate mental health as early as the first year. This educational strategy would help in preparing clinical associates to provide holistic care from an early stage in their training. As frontline health professionals, it would assist them to detect the contribution of mental health to a range of presentations and conditions and enable them to manage these co-morbidities. While there may be limitations as to how mental health content can be feasibly included in the earlier years, it may be possible to introduce them to mental health history taking, the mental status examination as well as the common mental disorders.

There is clearly a gap at University C with only two formal lectures on mental health compared to University A who has a week of formal teaching on campus and University B who has an extended period of facility-based formal teaching. The lack of formal teaching at University C might explain why an interviewee pointed out the lack of guidance as an issue as the students need to determine what is important on their own. While two lectures are clearly insufficient, some argument can be made to rationalise the conditions that are included in the curricula at all three universities. The utility of including uncommon disorders that clinical associates are unlikely to encounter or would not be required to manage is questionable. Rather, burden of disease data and prevalence data provide guidance. Results from the Global Burden of Disease Study 2017 suggest that depressive disorders, anxiety disorders, substance use disorders and schizophrenia form the prime inclusions as the mental illnesses that rank within the twenty leading causes of years with disability for males and females [34]. South African prevalence data suggest anxiety, mood, and substance use disorders are disorders that health workers are likely to encounter [35].

With respect to facility-based training for mental health, each university uses a different approach. The approach adopted by University A ensures students get practical exposure to a mental health unit and thus guarantees a wider variety of mental disorders. University B uses a more theoretical approach to their psychiatric block with very limited practical exposure which is usually coincidental. University C uses a number of sites with considerable variation in training depending on where a student is placed and distinct lack of standardisation compared to Universities A and B. As acknowledged by the interviewees at University A, the two-week mental health rotation is short (but guaranteed) but ideally needs to extended. Universities B and C allocate more time to mental health but most of the practical learning is by chance. Spending at least some of the time (e.g. two weeks) allocated to mental health in a hospital with a mental health unit would guarantee opportunities to learn.

Clinical associates’ scope of practice includes taking a history, performing an examination, performing diagnostic procedures, formulating a diagnosis, developing a management plan and performing specified procedures under supervision [28]. The list of procedures includes “Mental health examination”, “Mental Health History”, “Mini Mental State (MMS) examination” and “Counselling - family /mental health”[28]. All three universities include these in their curricula but the concern is the authentic application of these in practice. There is no indication in the literature reviewed on what an ideal split between mental health theory and practice would be for this cadre. It was notable that no evidence was found of suicide risk assessment being covered given South Africa’s high suicide rate [6]. In comparison, Canada includes suicide assessment in the competency profile of their physician assistants [36]. While all three universities utilise procedure or log books, only one has set specific experiential targets. Setting specific targets for mental health would ensure adequate practical exposure and help identify facilities where the opportunities to learn are too limited or where supervision is inadequate.

It is not clear how well the mental health training of South African clinical associates compares to equivalent cadres in other countries as information is not readily available. Though psychiatry is included in the competency profile of Canadian physician assistants, there is no indication of the duration of the mental health training they should receive [36]. Similarly, it is not clear how much mental health training Ethiopian health officers receive, but it is likely to be limited given that mental health services have not been a priority until recently [37]. A situational analysis of clinical officers (and nurses) working in primary care in Kenya found they had only a small amount of basic training in mental health [38]. A survey of non-specialist health workers in Malawi which included medical assistants found just 12% had received training in mental health [39]. Training in psychiatry varies widely between American physician programmes with some programmes offering comprehensive didactic teaching and clinical training while others offer only a small amount of didactic teaching with limited clinical opportunities in primary care settings [40]. The United Kingdom physician associate curriculum requires a minimum of only 90 hours in psychiatry out of 3 200 hours of teaching [41]. It is not clear how much of this is practical though their training does involve problem-based learning sessions, role-play scenarios and mental health consultations during general practice clinical placements [42].

While task sharing in mental health in South Africa has tended to focus on counselling interventions delivered by community health workers or lay health workers, the clinical training and background of clinical associates offers different possibilities at district hospitals and at primary care level. These include assessment and diagnosis, referral to higher levels of care when indicated, management of co-morbid mental and physical illness, and the prescribing of pharmacological treatment in addition to counselling interventions. There is evidence to support the involvement of primary health professionals in the collaborative care of adults with common mental disorders as well as primary health professional-led or collaborative care of adults with severe mental disorders and training should, therefore, be strengthened with respect to depression, anxiety and schizophrenia in order to allow clinical associates to deliver evidence-based interventions at primary level [17]. Improving clinical associates’ ability to detect and treat depression would also be critical in suicide prevention [43, 44]. All three universities offer some basic counselling training. Strengthening this component of their training may not only enable clinical associates to provide evidence-based counselling interventions such as problem solving for depression [45, 46] or motivational interviewing for substance use [47] themselves but may also enable them to supervise lay counsellors.

Qualified clinical associates may only be able to play a limited role in mental health service provision currently given the training gaps identified. However, strengthening of the mental health component of the three undergraduate programmes could see that role broadened for future graduates. Clinical associates who have already graduated may benefit from short courses to close some of the gaps. An advanced qualification such as Honours degree and/or a clinical specialisation for clinical associates in mental health should be considered given the concerning high prevalence of mental health disorders. The researchers intend to conduct further research to inform a conceptual framework for the provision of mental health services by clinical associates.

### Limitations

We purposively sampled participants from the three programmes to provide us with a comprehensive overview of their mental health training programme. However, we did not include participants from each of the training sites and there may be discrepancies between individual training sites and our global findings for the programme e.g. certain competencies that were covered in a particular programme may not have been covered at a particular site. All three programmes had been impacted by COVID-19. While we tried to elicit a description of the programmes pre-COVID-19 and during COVID-19, this was not possible with all participants. The document review was limited by what was submitted by BCMP/BMCP co-ordinators.

## Conclusion

The mental health training received by clinical associate students in SA varied between the three training programmes as well as within programmes with a number of areas identified for potential improvement. We recommend a model that integrates mental health as early as possible in the curriculum to maximise learning opportunities, focuses on common conditions and those that that contribute substantially to disease burden, includes compulsory rotations in mental health units, provides facility-based trainers with detailed guidance to standardise teaching across training sites, and includes specific experiential targets for the number of mental health procedures that are monitored using paper or electronic logbooks. Strengthened undergraduate clinical associate training programmes in mental health will provide the potential for their utilisation in task-sharing approaches in mental health services in SA.

## Data Availability

The dataset generated and analysed for this study are not publicly available in order to protect the confidentiality of the participants but are available from the corresponding author on reasonable request.

## Abbreviations

BCMP: Bachelor of Clinical Medical Practice.
BMCP: Bachelor of Medicine in Clinical Practice.
DALYs: Disability-adjusted life years.
NIDS: National Income Dynamics Survey.
SA: South Africa.
WHO: World Health Organization.
